# Randomized trial of telephone versus in-person delivery of a brief psychosocial intervention in post-stroke depression

**DOI:** 10.1186/s13104-017-2819-y

**Published:** 2017-10-10

**Authors:** Catherine J. Kirkness, Kevin C. Cain, Kyra J. Becker, David L. Tirschwell, Ann M. Buzaitis, Pamela L. Weisman, Sylvia McKenzie, Linda Teri, Ruth Kohen, Richard C. Veith, Pamela H. Mitchell

**Affiliations:** 1 Biobehavioral Nursing and Health Informatics, University of Washington, Box 357266, Seattle, WA 98195-7266 USA; 2 Biostatistics and School of Nursing, University of Washington, Box 357232, Seattle, WA 98195-7232 USA; 3 Neurology, University of Washington, Box 359775, Seattle, WA 98185-9775 USA; 4 UW Medicine, University of Washington, Box 359556, Seattle, WA 98195-9556 USA; 5 University of Washington School of Nursing, Box 357266, Seattle, WA 98195-7266 USA; 6 Psychosocial and Community Health, University of Washington, Box 357263, Seattle, WA 98195-7263 USA; 7 Psychiatry and Behavioural Sciences, University of Washington, Box 356560, Seattle, WA 98195-356560 USA; 8 Biobehavioral Nursing & Health Systems, University of Washington, Box 357260, Seattle, WA 98195-7260 USA

**Keywords:** Behavioural therapy, Psychosocial intervention, Depression, Randomized controlled trial, Stroke, Nurse therapist

## Abstract

**Background:**

A psychosocial behavioral intervention delivered in-person by advanced practice nurses has been shown effective in substantially reducing post-stroke depression (PSD). This follow-up trial compared the effectiveness of a shortened intervention delivered by either telephone or in-person to usual care. To our knowledge, this is the first of current behavioral therapy trials to expand the protocol in a new clinical sample. 100 people with Geriatric Depression Scores ≥ 11 were randomized within 4 months of stroke to usual care (N = 28), telephone intervention (N = 37), or in-person intervention (N = 35). Primary outcome was response [percent reduction in the Hamilton Depression Rating Scale (HDRS)] and remission (HDRS score < 10) at 8 weeks and 12 months post treatment.

**Results:**

Intervention groups were combined for the primary analysis (pre-planned). The mean response in HDRS scores was 39% reduction for the combined intervention group (40% in-person; 38% telephone groups) versus 33% for the usual care group at 8 weeks (p = 0.3). Remission occurred in 37% in the combined intervention groups at 8 weeks versus 27% in the control group (p = 0.3) and 44% intervention versus 36% control at 12 months (p = 0.5). While favouring the intervention, these differences were not statistically significant.

**Conclusions:**

A brief psychosocial intervention for PSD delivered by telephone or in-person did not reduce depression significantly more than usual care. However, the comparable effectiveness of telephone and in-person follow-up for treatment of depression found is important given greater accessibility by telephone and mandated post-hospital follow-up for comprehensive stroke centers.

*Clinical Trial Registration* URL: https://register.clinicaltrials.gov, unique identifier: NCT01133106, Registered 5/26/2010

**Electronic supplementary material:**

The online version of this article (doi:10.1186/s13104-017-2819-y) contains supplementary material, which is available to authorized users.

## Background

It is now well established that depression is a significant risk factor for having a stroke and also complicates recovery from stroke [1]. Further, meta-analyses show that roughly 30% of people with strokes suffer from clinical depression [2]. We previously showed that a brief psychosocial behavioural intervention delivered in-person by psychosocial nurse practitioners to community dwelling ischemic stroke survivors is efficacious in reducing depressive symptoms rapidly and sustaining that reduction over time [3]. At the time we began the previous study, Cochrane Database reviews showed few adequately designed studies of psychosocial and non-pharmacologic interventions, with relatively small effects [4, 5]. Our previous study was cited as one of those in progress that might add to support for such interventions [5].

Since the publication of the Cochrane review, our initial study, living well with stroke (LWWS) brief psychosocial intervention demonstrated significant effects for remission immediately following treatment and at 1 year compared to usual care (odds ratio 4.8, p 0.001 immediate post treatment; odds ratio 2.7, p 0.03 at 1 year) [3]. An additional three studies with similar psychosocial and behavioural interventions for post-stroke depression demonstrated efficacy in samples ranging from 24 to 188 stroke survivors [3, 6–8].

We are now reporting, according to CONSORT guidelines [9], a follow up randomized controlled trial (RCT) to compare a shorter version of the brief psychosocial behavioural intervention delivered by telephone or in-person to usual care. We also expanded recruitment to include persons with either ischemic or haemorrhagic stroke.

## Methods

### Aim

The primary aim was to test whether the brief intervention delivered in-person or by telephone is superior to usual care in terms of post-stroke depression treatment response (percent reduction in depressive symptoms) or remission in the short-term (immediately following treatment) and at 1 year post treatment.

### Design

Living well with stroke 2 (LWWS 2) was a randomized controlled efficacy study comparing two modes of delivery of a brief psychosocial behavioural intervention with usual care in both ischemic and haemorrhagic stroke survivors. It was designed as a three arm study with a pre-planned combining of the two intervention arms for primary analysis if they were found to be statistically equivalent. The Institutional Review Board of the University of Washington (Human Subjects Division) approved this study for protection of human subjects.

### Participants

One hundred people who were within 4 months of an ischemic or haemorrhagic stroke (verified by Computerized Tomography or Magnetic Resonance Imaging) consented to participate in this RCT. Clinical depression was identified by the screening score of the Geriatric Depression Scale [12] and verified at entry to the trial by the Diagnostic Interview and Structured Hamilton, previously validated as consistent with criteria from the 4th Edition of the Diagnostic and Statistical Manual of Mental Disorders [10].

### Protocol

This study followed the same recruitment and treatment protocols as described in living well with stroke 1 (LWWS 1) [3, 11]. The primary difference from LWW1 was the mode of delivery—offering treatment by telephone as well as in-person, and a reduction in the length of treatment (6 weeks vs 8 weeks). The treatment content remained the same and is described briefly.

### Screening for eligibility

**Fig. 1:**
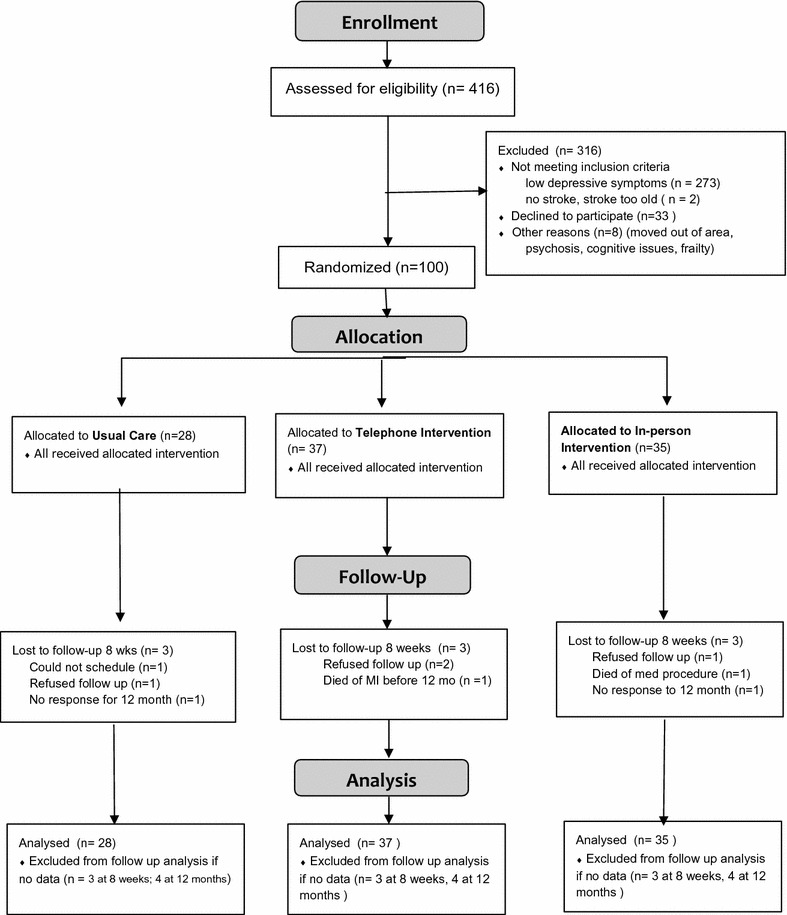
CONSORT flow diagram: LWWS 2

Patients were recruited from six university and community hospitals in the Seattle, WA area. Consecutive patients newly admitted with stroke were introduced to the study by intermediaries. Study research nurses obtained written consent from those who agreed to be screened for eligibility and administered the 30 item Geriatric Depression Scale [12]. Those who scored ≥ 11 were invited to join the full study. Clinical depression was verified as noted above. All screening and full study data were collected after the participant’s discharge from the hospital. Recruitment occurred from May 2010 to December 2014 and follow-up from August 2010 to December 2015. The trial ended in December 2015 when the last follow up was completed. The study flow chart is shown in Fig. 1.

A letter identifying each person’s participation in the study, but not their treatment allocation, and recommended antidepressant therapy was sent to the participant’s primary provider. In addition, all participants received written materials from the American Stroke Association about stroke recovery, including information on depression. All participants received ongoing medical care from their own provider, including antidepressant adjustment as determined by their provider.

### Brief psychosocial–behavioural intervention

Those randomized to intervention (either telephone or in-person) had one in-person orientation session with the psychosocial nurse practitioner therapist, either in their home or at our study offices. They received the participant manuals, discussed goals and expectations of each session, and learned how to fill out the homework sections.

Participant manuals were revised from those used in LWWS1 to reflect fewer sessions, based on a pilot with people who had completed LWWS1 control group follow up. The content of the intervention was adapted from the Seattle Protocols [13] in which cognitive behavioural therapy was conducted with adults with mild dementia. It was designed to challenge negative cognitions, reduce distortions and enable more adaptive ways of viewing specific situations or events. A behavioural intervention, used with adults with more moderate or severe dementia, increased the level of positive activities and decreased negative ones. Problem-solving was taught to provide effective strategies for behaviour change [13]. LWWS was revised from the Seattle Protocols for people with PSD, and aimed to reduce depression through pharmacotherapy of serotonin pathways, boosted by challenging negative cognitions and behaviours, enhancing engagement in and perception of positive events, and immediate provision of social support. LWWS2 content was unchanged in that we taught participants about the relationship of depression and stroke, that depressive symptoms are observable and potentially modifiable behaviours and that these behaviours and associated negative cognitions can be changed through observation and interaction. Participants were helped to identify activities that they found pleasant to do and to build these into their daily activities. Problem solving approaches were used to tailor this treatment to the circumstances of each participant and the challenges each faced in stroke recovery. This content was outlined in our prior publications and the manuals are available from the corresponding author [3, 11].

Following the in-person orientation session, each of the subsequent six sessions occurred either in-person, usually at the participant’s home, or by telephone. Topics were as follows: (1) introduction to behavioural therapy for depression after stroke, pleasant events; (2) scheduling pleasant events: problems and planning; (3) managing depression behaviours: problem-solving techniques; (4) changing negative thoughts and behaviours; (5) problem-solving in depth; (6) review of skills, generalization & strategies for maintenance of skills. At the end of each session, the participant provided an evaluation of the session and a rating of mood on a 0 (very happy) to 9 (very depressed) scale. Session length ranged from 10 to 80 min, with the telephone sessions somewhat shorter than the in-person ones (average 26 min versus 38 min).

As in LWSS 1, a family member or informal caregiver could opt to participate and provide data with the participant’s agreement. Fourteen family caregivers of the 72 participants in the intervention groups chose to be part of the study. One session included content directed particularly at the caregivers.

Sessions were recorded with the participants’ consent and treatment integrity was evaluated by listening to 10% of the recordings, in conjunction with the session checklist. No problems were detected over the course of the study (Additional file 1).

Participants in the intervention arms saw their primary care or stroke provider for stroke follow up care and were provided antidepressants as prescribed by their providers. Since antidepressant treatment is an informal standard of care in the community where this study place, we chose to have antidepressants prescribed by the participant’s medical care provider, rather than by the therapist in our study. The letter sent to providers by the principal investigators and the study psychiatrist stated: “The drugs that are recommended in the literature for post-stroke depression include the serotonin selective reuptake inhibitors (SSRI) sertraline as the primary antidepressant medication and citalopram or paroxetine as alternative medications for those who do not respond to sertraline. These SSRIs have been used extensively in at-risk cardiovascular populations without adverse cardiac effects.”

### Usual care control

Participants randomized to usual care reported on their progress at follow up visits in their homes from the research nurses at 8, 21 weeks, and 12 months following entry to the study. Seven family caregivers joined the 28 participants in this arm. As with our prior work we did not provide a “time and attention” control since the studies from which LWWS was adapted and other problem-solving interventions have not shown active control to be equivalent to active intervention [14]. Antidepressants were prescribed by the participant’s usual care provider in the same manner as they were for participants in the intervention arms.

#### Study timetable and assessments

Baseline data consisted of demographics, medical history, stroke characteristics, National Institutes of Health Stroke Scale score [15], Geriatric Depression Scale [12], Diagnostic Interview and Structured Hamilton Rating Scale for Depression [10], Barthel Index [16], Stroke Impact Scale [17], and percent perceived recovery (overall stroke impact) [17]. All were assessed at entry before randomization. Measures of depressive symptoms, stroke impact, and perceived recovery were assessed at 8 weeks (just following the intervention period), at 21 weeks and at 12 months following entry. Percent reduction in HDRS and remission data from the 8 week and 12-month time points were the four primary endpoints used to evaluate the primary aim.

#### Randomization and masking

The algorithm used for randomization was a modification of the minimization method described on page 84 of Pocock [18]. The algorithm was based on an imbalance score which measured, for a given set of random assignments, how far out of balance the study would be within strata for each factor and then summed over factors [19]. When a new subject was available for randomization, we computed what the imbalance score would be if this subject were assigned to usual care, or to telephone intervention, or to in-person intervention. Then a randomization was done to allocate two intervention participants to each control with each new assignment having a higher probability of less imbalance. The schema did not require equal numbers in each arm.

This procedure balanced the three groups with respect to important predictors of outcomes, including stroke severity (National Institutes of Health Stroke Scale—NIHSS), severity of baseline depression (Hamilton Rating Scale for Depression—HRSD), age, gender, and type of stroke (ischemic or haemorrhagic) (see Table 2). The study statistician generated the algorithm, which was securely stored and accessible only by the statistician and research nurse supervisor. Research nurses entered relevant assessment data and the research nurse supervisor informed the participant of the study arm to which they were randomized, thus masking outcome assessors to the participant’s randomization status. Participants were asked not to reveal their study arm to the outcome assessors. We did not detect any breaches in masking.

#### Sample size and statistical analysis

Our original recruitment plan called for 75 in each of the three arms, providing a power of 92% to detect an odds ratio of 2.7 between either intervention and control or between the two intervention groups. This was the odds ratio found in our initial study at the primary endpoint. We planned to combine the two intervention groups if there was no significant difference between them to increase the power to detect a difference between combined intervention and control. A lower rate of sadness and higher than previous rate of refusal led to a necessary reduction in the sample size. We reprogrammed our randomization algorithm to allocate remaining recruits on a ratio of 2 in-person, 2 telephone, 1 usual care. Assuming a total final sample of 165, this reduced power to 74% for an odds ratio of 2.7 and 80% for an odds ratio of 2.9. Continued low enrollment reduced our final sample to 100, which an interim analysis had shown as having similar effect size with our original estimate.

Analysis of outcomes was conducted per protocol and without imputation. Percent reduction in HRSD was the primary symptom response endpoint, as well as remission, defined here as HRSD score ≤ 10 (no longer meeting depression criteria) [20, 21]. We used analysis of variance for continuous variables (percent reduction in HRSD) and logistic regression for binary variables (remission or not).

## Results

We tracked more than 1000 consecutive stroke inpatients for screening eligibility, with 416 consenting to be screened for the study. As shown in Fig. 1, 141 were eligible and 100 agreed to full enrollment. Figure 1 shows reasons for exclusion. Nine participants were lost to follow-up by the first outcome assessment at 8 weeks, with an additional three dropping out by the 12-month assessment. Loss to follow-up was equal in all three groups and reasons are shown in Fig. 1. Two participants died and both deaths were attributed to underlying cardiocerebrovascular disease and not to study participation. No harms attributable to the study were identified.

Tables 1 and 2 show demographic and stroke/health characteristics. These were comparable at baseline for all three groups in LWWS 2 except for a lower prevalence of diabetes and lower history of depression in the usual care control group. The sample was evenly divided by gender, with the majority being white or of more than one race. Ages ranged from 23 to 88 years, with a mean of 60 years. Over 70% of each group had a history of one or more past episodes of depression, and over 40% were taking an antidepressant when they entered the study, with the lowest percentages of these in the control group. The demographics and stroke characteristics are consistent with those of stroke survivors in the United States [22].

**Table 1:** Baseline demographic characteristics—LWWS 2

Characteristic	Telephone (N = 37)	In-person (N = 35)	Control (N = 28)
Gender			
Male N, %	19 (51.4%)	17 (48.6%)	14 (50%)
Female N, %	18 (48.6%)	18 (51.4%)	14 (50%)
Age, years (mean, range)	61.7 (31–85)	58.5 (23–83)	60.7 (32–88)
Marital status N, %			
Single	3 (8.1%)	8 (22.9%)	2 (7.1%)
Married, partnered	22 (60.4%)	15 (42.9%)	19 (67.9%)
Widowed, divorced, separated	12 (32.4%)	12 (34.3%)	7 (25%)
Current living arrangement N, %			
Homeless	0	1 (2.9%)	0
Alone	13 (35.1%)	6 (17.1%)	4 (14.3%)
With spouse, partner	20 (54.1%)	15 (42.9%)	16 (57.1%)
With relatives, others	3 (8.1%)	9 (25.7%)	4 (14.3%)
Group housing	1 (2.7%)	4 (11.4%)	4 (14.3%)
Race, ethnicity N, %			
Hispanic ethnicity	1 (2.7%)	1 (2.9%)	3 (10.7)
More than one race	4 (10.8%)	8 (22.9%)	2 (7.1%)
White only	30 (81.1%)	25 (71.4%)	24 (85.7%)
Black only	2 (5.4%)	2 (5.7%)	1 (3.1%)
Asian only	1 (2.7%)	0	1 (3.1%)

**Table 2:** Baseline stroke and health characteristics comparing LWWS 1 and 2

Characteristic	Telephone LWSS2 (N = 37)	In-person LWSS2 (N = 35)	Control LWSS 2 (N = 28)	In-person LWSS 1 (N = 48)	Control LWSS 1 (N = 53)
NIHSS score, mean (SD, range) (Median, IQR)	3.4 (3.4, 0–15) 2 (1–5)	3.4 (3.6, 0–14) 2 (1–5)	3.5 (3.8, 0–12) 2 (1–6)	6.08 (4.4, 0–17)	6.21 (5.05, 0–17)
HRSD, mean (SD, range)	18.0 (3.1, 12–26)	19.1 (3.2, 14–27)	18.3 (2.9, 13–23)	20.0 (4.53, 10–29)	19.8 (4.15, 11–29)
Barthel index, mean (SD) (Median, IQR)	91.8 (17.5) 98 (92–98)	89.1 (20.3) 97 (89–97)	88.0 (22.6) 97 (87–97)	81.9 (23.2)	83.5 (22.3 SD)
Perceived percent recovery, mean (SD, range)	61.6 (22.8, 0–95)	60 (25.9, 2–95)	65.9 (24.9, 10–95)	46.3 (23.1, 0–90)	55.3 (19.7, 10–100)
History of depression (number, %)	32 (86%)	27 (77%)	20 (71%)	36 (75%)	37 (69.8%)
Currently taking antidepressant medication	19 (51%)	16 (46%)	12 (43%)	29 (60.4%)	34 (64.2%)
Ischemic stroke (includes ischemic with H conversion N, %)	32 (86%)	31 (89%)	22 (79%)	48 (100%)	53 (100%)
Intraparenchymal haemorrhagic stroke	4 (11%)	4 (11%)	3 (11%)	0	0
Subarachnoid haemorrhage	1 (2%)	0	3 (11%)	0	0
Current antihypertensive medication	26 (70%)	28 (80%)	20 (71%)	39 (81.3%)	40 (75%)
Heart failure	0	6 (17%)	2 (7%)	10.4%	13.2%
Diabetes	11 (30%)	12 (34%)	5 (17%)	16.7%	43.4%

*SD* standard deviation, *IQR* interquartile range

The comparison of clinical characteristics for LWWS 1 and 2 shown in Table 2 indicates that participants in LWWS2 had less severe strokes, were less depressed, perceived themselves as more recovered and had a lower prevalence of heart failure and diabetes than did the LWWS 1 participants.

By the first post-treatment time point 46% of usual care, 53% of telephone intervention, and 61% of in-person intervention participants were taking an antidepressant. The proportion of people taking antidepressants in each group remained roughly the same until the end of the study (12 months). At the first post-treatment time period 27% of the control group, 6% of the telephone intervention group and 16% of the in-person intervention group sought counselling outside the study. By the 12-month time point, 20% of controls, 9% of telephone and 23% of in-person participants were using outside counselling.

Table 3 shows response to treatment and remission. Since there was no significant difference in response between the telephone and in-person intervention conditions, the intervention groups were combined as pre-planned. The percentage decrease in HRSD scores and percentage of participants in remission were both greater in the combined intervention groups than in the usual care control at the first post-treatment time point, but the difference was not statistically significant.

**Table 3:** Response to treatment (change in depressive symptoms) LWWS 2

Variable	Control	Confidence interval	Combined intervention	Confidence interval	F	p (combined intervention vs control)*
Depressive symptoms (HDRS) percent change over time—primary endpoints shaded						
HDRS % change 8 weeks (mean, SD)	(N = 26) − 33.2 (22.5)	− 42.3 to − 24.1	(N = 65) − 39.3 (26.4)	− 45.8 to − 32.7	1.07	0.304
HDRS % change 21 weeks (mean, SD)	(N = 25) − 38.5 (28.3)	− 50.2 to − 26.9	(N = 63) − 40.1 (29.5)	− 47.6 to − 32.7	0.05	0.82
HDRS % change 12 months (mean, SD)	(N = 25) − 39.0 (25.1)	− 49.4 to − 28.7	(N = 63) − 42.1 (31.9)	− 50.1 to − 34.1	0.19	0.67

Time point	Control (N, %)	Combined intervention (N, %)	Odds ratio	Confidence interval	p combined intervention vs control**
Remission (HDRS ≤ 9) over time LWWS2—primary endpoints shaded					
8 weeks (T2) N = 91	7/26 (27%)	24/65 (37%)	1.6	0.58 to 4.33	0.36
21 weeks (T3) N = 88	9/25 (36%)	25/63 (40%)	0.6	0.45 to 3.1	0.75
12 months (T4) N = 88	9/25 (36%)	28/63 (44%)	0.7	0.55 to 3.7	0.47

* Anova combined intervention versus usual care control, NS at all time points

** Odds ratio combined intervention versus usual care control: NS at all time points

Preplanned exploratory analyses showed there were no significant interactions with age, gender, severity of stroke, severity of depression, use of antidepressants or use of outside counselling. Sensitivity analyses imputing a range of best and worst values for the missing follow up data did not change results from the planned complete case analysis.

**Fig. 2:**
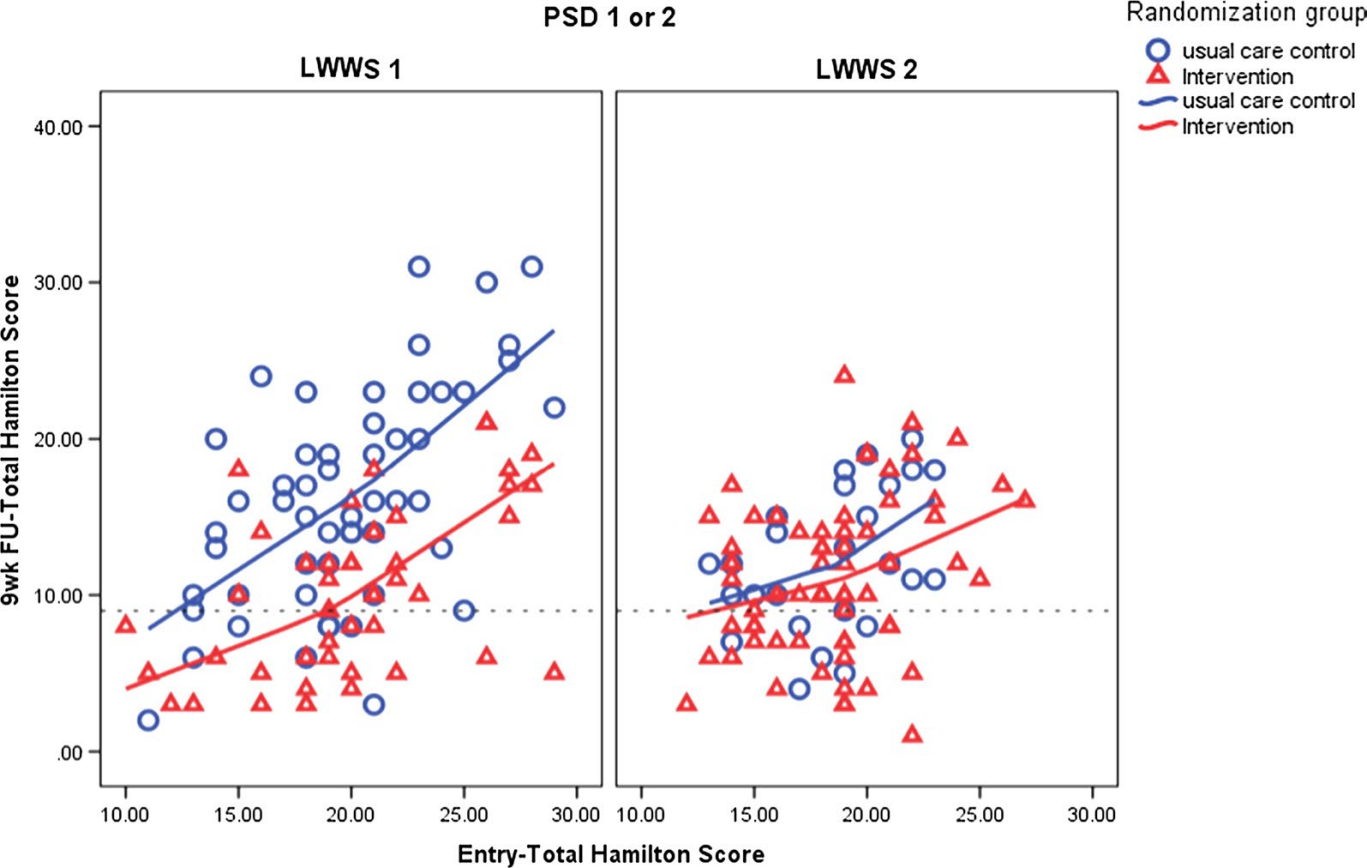
Comparison of response to treatment LWWS 1 and LWSS 2. The dotted line is the HRSD score indicating remission

When compared to LWSS1, there was less improvement in depressive symptom scores for LWWS 2 and more improvement in the usual care group at the first follow up (immediately after treatment) with no significant difference in percent change in HRSD between studies after that. This comparison is shown in Fig. 2.

## Discussion

While the intervention effect across time in LWWS 2 was weaker than that in LWWS 1, this should not be cause for dismissing the value of a brief psychosocial behavioural strategy in reducing depressive symptoms in stroke survivors. First, there was a clinically important and equivalent overall reduction of depressive symptoms and remission in both the telephone and in-person intervention groups in LWWS 2. In contrast to LWWS 1, there was more rapid improvement in depression among the control group in LWWS 2, although the rate of remission at 12 months was still lower than seen in intervention groups. Second, the intervention produced no harm.

It could be argued that shortening the intervention by 2 sessions accounted for the reduced effect size in LWWS2. However, the session mood ratings for both LWWS1 and 2 showed improvement by session 3 and 4 for those intervention participants who had early remission, (data not shown) reinforcing our assumption that the content remained the same. However, the fact that up to 23% of the intervention participants sought outside counselling by one year after the intervention ended may have reflected their desire for more sessions (data not shown).

Differences in the baseline stroke and depression characteristics of participants in LWWS 2, when compared with LWWS 1, may have contributed to the differing rapidity and degree of improvement in depression. First, the strokes in LWWS2 were overall less severe, as indicated by the lower average NIHSS scores for both intervention and control participants (Table 2). That the strokes were less severe may reflect temporal changes with improvement in rapidity of treatment and greater use of intravenous thrombolytic therapy and the use endovascular clot retrieval treatment for ischemic stroke nationwide [23, 24]. The use of antidepressants at baseline was lower in LWSS 2, but there was a higher percentage of participants with prior history of depression than in LWSS 1. Twenty-seven percent of the control participants sought outside counselling on their own, which may have mimicked the effects of our intervention and reduced the ability to detect a strong intervention effect. Finally, we cannot discount the possibility that the follow up questionnaires regarding mood and stroke recovery constituted a reflective therapy in themselves. This effect has been reported in other intervention trials targeting behaviour in both clinical and nonclinical populations [25].

While we did not replicate the persistent strength of response to the intervention versus control in this second trial, our findings do not negate the value of psychosocial interventions. The published trials of psychosocial and behavioural intervention for PSD all show improvement in depressive symptoms compared to controls in over 300 participants, including those who are aphasic [3, 6–8].

Experience with psychosocial interventions in other disorders suggest two directions for further study. First, a meta-analysis of psychosocial trials including the moderating variables might provide better guidance regarding the personal, medical and intervention characteristics that best predict the efficacy of psychosocial interventions in PSD [26]. Second, a test of the feasibility and acceptability of incorporating LWWS into busy Comprehensive Stroke Center outpatient clinics could provide the basis for a pragmatic multi-site trial of brief interventions for PSD.

### Limitations

The most important limitation of this study was the small sample size from a limited geographic area. Although the study was powered appropriately to detect an effect similar to our first study with its small sample, the more modest effect we actually achieved negated our ability to demonstrate a statistically significant difference from the control group. While the similarity of this sample to the characteristics of stroke survivors in the US suggests the possibility of generalizability to a wider group, a much larger national sample would be needed for convincing results. Finally there were unadjusted multiple comparisons of the primary outcomes (two response measures at two times), raising the possibility of Type 1 error and possible over-statement of the effects of the intervention.

## Conclusions

Replication and adaptation of a brief psychosocial behavioural intervention for post-stroke depression showed that providing the intervention by telephone was as effective as conducting it in-person. The response, while clinically important and favouring intervention groups over usual care, was not statistically significant. These finding nevertheless provide confidence for continue recommendation of psychosocial and behavioural treatment to stroke survivors who are depressed, for using the telephone to provide this treatment, for testing the incorporation of this treatment in everyday practice, and ultimately a pragmatic multi-site trial.

## Additional file

**Additional file 1.** Consort check list.

## Abbreviations

CONSORT: Consolidated Standards of Reporting Trials
HRSD: Hamilton Rating Scale for Depression
LWWS 1, 2: living well with stroke trials 1 and 2
NIHSS: National Institutes of Health Stoke Scale
PSD: post-stroke depression
RCT: randomized controlled trial
SSRI: serotonin selective release inhibitor
